# Phytochemical Screening, Antioxidant and Antibacterial Activities of Pollen Extracts from *Micromeria fruticosa, Achillea fragrantissima,* and *Phoenix dactylifera*

**DOI:** 10.3390/plants10040676

**Published:** 2021-04-01

**Authors:** Omar Sadeq, Hamza Mechchate, Imane Es-safi, Mohamed Bouhrim, Fatima zahra Jawhari, Hayat Ouassou, Loubna Kharchoufa, Mashail N. AlZain, Nurah M. Alzamel, Omkulthom Mohamed Al kamaly, Abdelhakim Bouyahya, Amina Benoutman, Hamada Imtara

**Affiliations:** 1 Faculty of Medicine, Arab American University Palestine, Jenin B.P. 240, Palestine; omar.sadiq@aaup.edu; 2 Laboratory of Biotechnology, Environment, Agri-Food, and Health (LBEAS), Faculty of Sciences, University Sidi Mohamed Ben Abdellah (USMBA), Fez B.P. 1796, Morocco; Imane.essafi1@usmba.ac.ma (I.E.-s.); jawhari.fatimazahra@gmail.com (F.z.J.); 3 Laboratory of Bioresources, Biotechnology, Ethnopharmacology and Health, Faculty of Sciences, Mohammed First University, Oujda B.P. 717, Morocco; mohamed.bouhrim@gmail.com (M.B.); hayatouassou@gmail.com (H.O.); khloubna43@gmail.com (L.K.); 4 Department of Biology, College of Sciences, Princess Nourah bint Abdulrahman University, Riyadh 11451, Saudi Arabia; mnalzain@pnu.edu.sa; 5 Department of Biology, College of Science and Humanities, Shaqra University, Shaqra B.P. 11961, Saudi Arabia; nalzamel@su.edu.sa; 6 Department of Pharmaceutical Sciences, College of Pharmacy, Princess Nourah Bint Abdulrahman University, Riyadh B.P. 11451, Saudi Arabia; omalkmali@pnu.edu.sa; 7 Laboratory of Human Pathologies Biology, Department of Biology, Faculty of Sciences, and Genomic Center of Human Pathologies, Faculty of Medicine and Pharmacy, Mohammed V University, Rabat B.P. 10045, Morocco; boyahyaa-90@hotmail.fr; 8 Laboratory of Biology, Environment, and Sustainable Development, Higher Normal School, Abdelmalek Essaadi University, Tetouan B.P. 2117, Morocco; aminabenoutman4@gmail.com; 9 Faculty of Arts and Sciences, Arab American University Palestine, Jenin B.P. Box 240, Palestine

**Keywords:** pollen, antioxidant, antibacterial, *Micromeria fruticosa*, *Achillea fragrantissima*, *Phoenix dactylifera*, pollen extraction

## Abstract

Pollen is a male flower gametophyte located in the anthers of stamens in angiosperms and a considerable source of compounds with health protective potential. In the present work, phytochemical screening was carried out as well as analysis of the antioxidant and antibacterial properties of pollen extracts from *Micromeria fruticosa*, *Achillea fragrantissima,* and *Phoenix dactylifera* growing wild in Palestine. Phytochemical screening examined the total flavonol, flavone and phenolic content. The DPPH (1,2-Diphenyl-1-Picrylhydrazyl) and FRAP (ferric reducing *antioxidant* power) methods were used to assess antioxidant propriety, and disc diffusion, minimum inhibitory and bactericidal concentration tests were used to test the pollen extract’s antibacterial activity against multidrug-resistant (MDR) clinical isolates. The highest level of total phenolic was found in the extract of *Micromeria fruticosa* (56.78 ± 0.49 mg GAE (Gallic Acid Equivalent)/g). The flavone and flavonol content of samples ranged from 2.48 ± 0.05 to 8.03 ± 0.01 mg QE (Quercetin Equivalent)/g. *Micromeria fruticosa* pollen with IC50 values of 0.047 and 0.039 mg/mL in the DPPH and FRAP assays, respectively, showed the greatest radical scavenging action. In addition, this pollen showed a mild antibacterial action against the microorganisms studied, with MICs varying from 0.625 to 10 mg/mL and inhibition diameters ranging from 13.66 ± 1.5 to 16.33 ± 1.5 mm.

## 1. Introduction

Medicinal plants’ traditional use has been investigated recently in order to scientifically approve their activities, confirm their usage and inspire the drug industry towards new safe and effective alternatives [1,2]. Today a large part of studied drug substances are natural products or inspired by a natural compound [3]. The application of natural products or bioinspired molecules covers a wide range of diseases relevant to recent public health problems, among them antibiotic-resistant bacteria and oxidative stress. 

Since the era of Alexander Fleming and its miracle discovery of penicillin, humanity has partially won the war against those microscopic germs, bacteria [4]. However, in terms of evolution and survival, they have developed with time a resistance oriented toward the commonly used antibiotics [5]. An alarming situation was declared by the World Health Organization stating that antibiotic-resistant bacteria are now causing a serious threat to global human health [6,7]. In this context, natural products are investigated as potential antimicrobial agents that can overcome the current resistance problem [8,9,10,11]. Natural products have also been investigated in other human health issues such as reactive oxygen species (ROS). This term has recently become well-known due to its involvement in several complications such as cardiovascular and lung diseases, certain forms of cancer, immune diseases, and inflammation [12]. As is known, ROS is both beneficial and detrimental to biological processes. Its action toward the physiological functions of several cellular reactions shows the positive effects of reactive oxygen species. Conversely, at high concentrations, reactive oxygen species can damage various cellular components such as nucleic acids, proteins, and lipids [13,14]. Several synthetic antioxidants have been proposed over the years for the prevention and treatment of certain diseases, but their toxicity has led to harmful effects in their use [15].

In this general context of recent interest in natural products as an effective solution to modern illnesses, especially to antibiotic resistance and reactive oxygen species, three plants were investigated, namely *Micromeria fruticosa, Achilleafra grantissima*, and *Phoenix dactylifera*.

*Micromeria fruticosa* is an aromatic herb from the Lamiaceae family [16], widespread in the eastern Mediterranean regions including Palestine, known as, Qurnya, Duqat das, and Ishbitesh-shai [17]. The aerial parts of *Micromeria fruticosa* (leaves, stalk, and flower) are used commonly in Palestinian society, as treatment of headache, abdominal pains, skin and eye infections, colds, and wounds [18,19]. The essential oil and extracts of *Micromeria species* have shown a great deal of antimicrobial and antioxidant biological activity [20]. It has been determined that different extracts of *Micromeria fruticosa* are potent DPPH radical scavengers compared to ascorbic acid [21]. In comparison, the *Micromeria fruticosa* ethanolic extract has demonstrated substantial antimicrobial activity against various tested microorganisms, even more than ampicillin [22].

*Achilleafra grantissima* is a flowering plant of the Asteraceae family, widely distributed in Northeastern Africa and the Middle East (Egypt, Jordan, Palestine, Syria, Lebanon, Iraq, and Saudi Arabia). *Achilleafra grantissima* is one of the most popular and important herbs of traditional Arabic medicine, used for medicinal purposes in Palestine to prepare medicinal teas to prevent and treat various health problems [23].

*Phoenix dactylifera,* known as the date palm, is one of the most popular plants in South Asia, the Middle East and North Africa [24], belonging to the Arecaceae family [25]. The date palm of Palestine has various nutritional and medicinal properties [26]. Generally, *Phoenix dactylifera* parts are used in folk remedies for the treatment of various diseases such as cough, rheumatism, nephropathy, gastropathy, respiratory infections, asthma, cancer and high blood pressure [27]. *Phoenix dactylifera* possesses many pharmacological properties such as antioxidant, hepatoprotective, anticancer, and gastroprotective antifungal activities. Moreover, the date fruit of *Phoenix dactylifera* has the ability to scavenge superoxide and hydroxyl radicals and to inhibit iron-induced lipid peroxidation and protein oxidation in the rat brain homogenate [28]. *Phoenix dactylifera* extract also demonstrated a protective effect against dimethoate-induced hepatic damage in rats [29]. Moreover, Ishurda and John (2005) have shown that the glucans prepared from date fruits exhibited a potent anticancer activity [30]. On the other hand, in vivo study showed that the aqueous and ethanolic nature of *phoenix dactylifera* increased the gastrointestinal transit time [31]. *Phoenix dactylifera* also possesses antifungal activity against *Candida albicans* [32]. 

Seeds, stems, leaves, fruits, flowers, and even entire parts of these plants have been used medicinally in the past. However, no specific research has been done on the pollen of these species. This research was conducted to provide information of phytochemical screening, antioxidant potential, and antibacterial activity of pollen extracts from Micromeria fruticosa, Achillea fragrantissima, and Phoenix dactylifera on the growth of two Gram-positive strains (Staphylococcus aureus and Streptococcus faecalis) and Gram-negative strains (Pseudomonas aeruginosa and Escherichia coli).

## 2. Results

### 2.1. Phytochemical Screening of Pollen Extracts of Selected Species from Palestine

Phytoconstituents detected in pollen extracts such as tannins, catechin tannins, gallic tannins, flavonoids, sterol, alkaloids, saponosides, cardiac glycosides, oses, holosides and mucilage are shown in Table 1. According to the results, tannins were detected in all pollen extracts in great quantities. Flavonoids, sterol and cardiac glycosides were also found in all pollen extracts. Catechin tannins, oses, and holosides were detected in all pollen extracts except for *Phoenix dactylifera*. Traces of saponosides were detected in a small amount in all pollen extracts except for *Phoenix dactylifera*. The Dragendorff test demonstrated the presence of alkaloids in all pollen extracts, while the Mayer test showed the absence of alkaloids. Mucilage was also absent. The results of phytochemical screening show that the pollen extracts of *Micromeria fruticosa* and *Achilleafra grantissima* were rich in phytochemical compounds compared to *Phoenix dactylifera*. 

### 2.2. Antioxidant Potential of Pollen Extracts of Selected Species from Palestine

**Figure 1:**
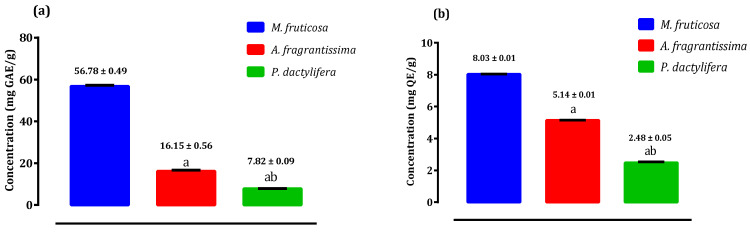
Total phenolic, flavone and flavonol content of pollen extracts of selected species from Palestine. (**a**): Total phenolic; (**b**): flavone and flavonol content. ^a^ *p* < 0.05 as comapred to pollen extract of *Micromeria fruticosa* in the same column; ^b^ *p* < 0.05 as compared to to pollen extract of *Achillea fragrantissima* in the same column.

Figure 1 demonstrates the estimation of the flavonol, flavone and total phenolic content of the pollen extracts from *Micromeria fruticosa*, *Achillea fragrantissima*, and *Phoenix dactylifera*. The results of total phenolic ranged from 7.82 ± 0.09 mg GAE/g in pollen of *Phoenix dactylifera* to 56.78 ± 0.49 mg GAE/g in pollen of *Micromeria fruticosa* (Figure 1a), while flavone and flavonol content ranged from 2.48 ± 0.05 mg QE/g in pollen of *Phoenix dactylifera* to 8.03 ± 0.01 mg QE/g in pollen of *Micromeria fruticosa* (Figure 1b).

### 2.3. Antioxidant Activity of Pollen Extracts of Selected Species from Palestine

**Figure 2:**
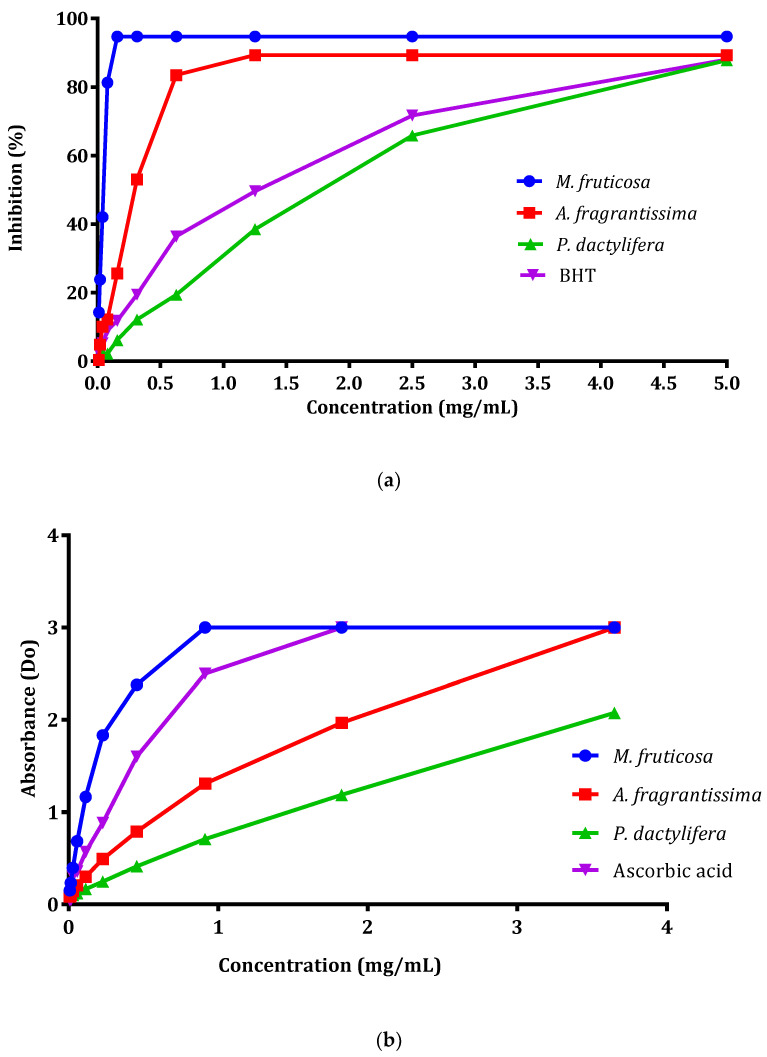
Antioxidant activities of pollen extracts of selected species from Palestine. (**a**) The DPPH radical-scavenging assay; (**b**) the reducing power assay (FRAP). *M. fruticosa*: *Micromeria fruticosa*; *A. fragrantissima*: *Achillea fragrantissima*; *P. dactylifera*: *Phoenix dactylifera*; BHT: Butylated hydroxytoluene.

The IC_50_ values of DPPH radicals scavenging for pollen extracts of *Micromeria fruticosa, Achillea fragrantissima,* and *Phoenix dactylifera* were 0.047, 0.295, and 1.820 mg/mL, respectively (Table 2). Pollen extracts from *Micromeria fruticosa* and *Achillea fragrantissima* scavenged DPPH free radicals more effectively than pollen extracts from *Phoenix dactylifera*, which demonstrated a moderate activity. Figure 2a illustrates the dose–response curves of the DPPH radical activities of pollen extracts from *Micromeria fruticosa*, *Achillea fragrantissima*, and *Phoenix dactylifera*. The IC_50_ values of ferric reducing antioxidant power of pollen extracts from *Micromeria fruticosa*, *Achillea fragrantissima*, and *Phoenix dactylifera*, are summarized in Table 2. The IC_50_ values were found to be 0.039, 0.248 and 0.585 mg/mL, respectively. The dose–response curves of FRAP radical activities of pollen extracts are shown in Figure 2b.

The antioxidant activity results indicate that the pollen extract from *Micromeria fruticosa* has the ability to quench free radicals in a concentration less than that of butylated hydroxytoluene (BHT) and ascorbic acid, which indicated that this extract was a good antioxidant, with radical scavenging activity.

### 2.4. Antibacterial Activity of Pollen Extracts of Selected Species from Palestine

**Figure 3:**
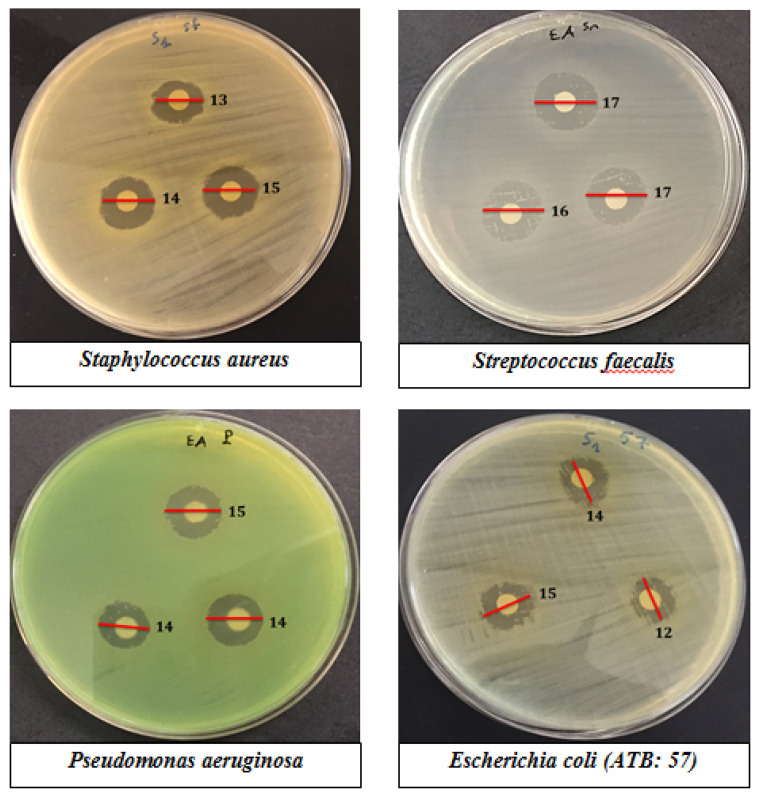
Diameters of the inhibition zones (mm) produced by pollen extracts in an AWD test against Gram positive and negative strains.

The antibacterial activity of the hydroalcoholic extract from these pollens was studied by the agar well diffusion assay method using the Gram-positive strains (*S. aureus*, *S. faecalis*) and the Gram-negative strains (*E. coli*, *P. aeruginosa*). The pollen extract of *Micromeria fruticosa* showed remarkable activity against pathogens tested with inhibition zones ranging from 13.66 ± 1.5 mm to 16.33 ± 1.5 mm (Table 3, Figure 3). The highest bacterial inhibition zone for the pollen extract of *Micromeria fruticosa* was observed against *S. faecalis* (16.33 ± 1.5 mm), followed by *staphylococcus aureus* (14.33 ± 0.6 mm), *P. aeruginosa* (15.66 ± 1.5 mm), and *E. coli* (13.66 ± 1.5 mm). The pollen extract of *Achillea fragrantissima* was active against *S. aureus* with an inhibition zone of 16.33 ± 0.6 mm, *P. aeruginosa* (15.33 ± 1.15 mm), and *S. faecalis* (14.33 ± 1.15 mm). The pollen extract of *Phoenix dactylifera* showed activity against *S. faecalis* (14.66 ± 0.6 mm) and *P. aeruginosa* (14 ± 1 mm). Ethanol (70%) was used as control and did not have any effect on all studied strains.

The MIC and MBC of the pollen extracts from *Micromeria fruticosa*, *Achillea fragrantissima*, and *Phoenix dactylifera* against the four pathogen agents tested were determined (Table 4). MIC values observed for these pollens were between 0.015 mg/mL and 10 mg/mL, and MBC were between 0.313 mg/mL and >10 mg/mL. 

### 2.5. Correlations Analysis

**Figure 4:**
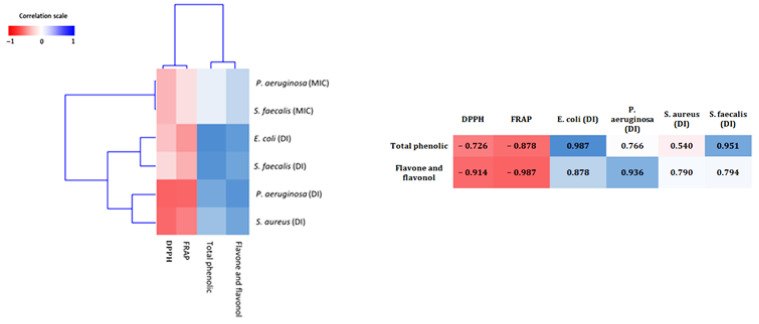
Heat map illustrating the correlation between flavone, flavonol and total phenolic and content with the assessed antioxidant and antimicrobial activities.

To better grasp the relationship between flavonol, flavone, total phenolic content and the assessed antioxidant and antimicrobial activities, a heat map model was used. This method replaces the values with colors; a red color corresponds to a negative correlation, a blue color corresponds to a positive correlation while the intermediate values are illustrated by the corresponding gradient between the two colors (Figure 4). There was a clear negative correlation between the overall flavonol, flavone, phenolic content and DPPH, FRAP IC50, and a positive correlation with antibacterial activity. 

## 3. Discussion

This is the first study on the phytochemical characterization of pollen extracts from *Micromeria fruticosa*, *Achillea fragrantissima* and *Phoenix dactylifera*. In the present study, the chemical compounds detected in pollen extract of *Micromeria fruticosa* were identical to those in the study carried out by Abu-Reidah et al. on the leaves of *Micromeria fruticosa*, in which more than 180 phytochemicals were reported (87 flavonoids, 41 phenolic acid, 16 terpenoids, eight sulfate derivatives, seven iridiodes, and others) [33]. For the pollen extract of *Achillea fragrantissima*, the presence of flavonoids, tannins, catechin tannins and gallic tannins were also reported (extract of plant in the flowering stage) [34], as well as for the sterol [35]. These compounds are known for their medical importance; for example, many studies have demonstrated the medicinal properties of the alkaloid compound as an antioxidant, anti-inflammatory, antimalarial, antimicrobial, anti-cytotoxicity and antispasmodic agent [36]. Steroids derived from plants are also widely used in medicines for their biological activities such as cardiotonic, antibacterial and insecticidal properties [37].

In addition, a study conducted on *Phoenix dactylifera* pollen showed its richness in tannins, catechin tannins, gallic tannins, flavonoids, sterol, oses, holosides, and alkaloid compounds [38]. In general, *Phoenix dactylifera* pollen consists of moisture (28.8%), ash (4.57%), crude fiber (1.37%), crude fat (20.74%), crude protein (31.11%), carbohydrate (13.41%), vitamins (A, E and C), minerals and amino acids [39].

Regarding the flavone, flavonol and total phenolic content, the pollen extract of *Micromeria fruticosa* contains significant amounts followed by *Achillea fragrantissima* and *Phoenix dactylifera*. These results agree with many studies that have identified the phenolic and flavonoid compounds in pollen [40,41], and they have been shown to have potent antioxidant properties [41,42]. Their antioxidant capacities support the human body’s battle against diseases by absorbing free radicals and chelating metal ions that could catalyze the production of reactive oxygen species (ROS), which facilitates lipid peroxidation [36]. In a previous study conducted by Bakour et al. to determine the phenolic/flavonoid compounds of 14 types of pollen using HPLC-DAD, the findings revealed that caffeic acid derivatives are present in the majority of pollen samples examined [41]. In a study conducted by El-Kholy et al. on phenolic compounds in *Phoenix dactylifera* pollen, the results also showed the presence of gallic acid, catechin, caffeic acid, rutin, quercetin, cinnamic acid, coumaric acid, ferulic acid, naringenin and propyl gallate [43].

The differences in total phenolic content of dates in the literature is the result of agronomical differences, season, soil type, variety, growing condition, maturity, fertilizer, genomics, climate, moisture content, methods of extraction, storage conditions and standards used [44,45]. Total phenolic of pollen extracts from *Micromeria fruticosa*, *Achillea fragrantissima,* and *Phoenix dactylifera* showed a positive correlation with flavone and flavonol content, while a negative correlation was observed with IC_50_ of DPPH and FRAP. Thus, the reducing activity of potential test extracts could be due to the existence of polyphenols and their hydrogen donating capacity, as reported by Shimada et al. [46]. Probably, these extracts contain a large concentration of reductone, which may react with radicals to stabilize and end radical chain reactions [47]. 

Regarding antibacterial activity, previously published reports report that extracts with MIC less than 100 µg/mL can be considered to have very good antibacterial activity [48]. According to these criteria, the hydroalcoholic extracts of pollen demonstrate significant activity against *S. faecalis* (MIC = 0.015–1.25 mg/mL; MBC = 0.313–1.25 mg/mL). Moderate activity of pollen extracts was observed against *S. aureus* and *P. aeruginosa* with MIC and MBC ranges of 5 and 5–10 mg/mL, respectively. The lowest activity of the hydroalcoholic extracts of these pollens was observed against *E. coli* with MIC = 10 mg/mL and MBC > 10 mg/mL. This can be explained by the highest resistance of Gram-negative bacteria due to the complexity of their cell wall, containing a double membrane as opposed to the unique glycoprotein/teichoic acid membrane of Gram-positive bacteria [49]. Preliminary phytochemical analyzes revealed that the pollen extracts from *Micromeria fruticosa*, *Achillea fragrantissima*, and *Phoenix dactylifera* contain tannins, flavonoids, saponosides, cardiac glycosides, sterols and alkaloids. These bioactive compounds are believed to have been used by plants and their parts to protect against bacteria and are responsible for antimicrobial activity [50,51]. A possible mechanism for this phytochemical activity may be either through inhibiting the growth of microbes, inducing cellular membrane perturbations, interference with certain microbial metabolic processes, or modulation of signal transduction or gene expression pathways. However, these mechanisms may all occur at the same time as a result of the synergistic effect between the compounds [52].

In this study, a positive correlation was also observed between flavonol, flavone and total phenolic content with antibacterial activity using a AWD assay. 

## 4. Materials and Methods

### 4.1. Species

**Figure 5:**
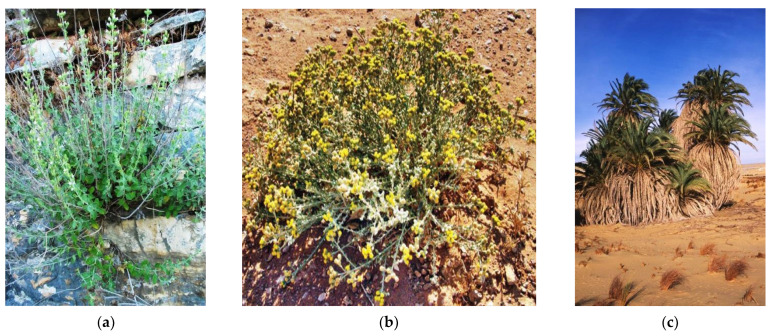
(**a**): *Micromeria fruticosa* (L.); (**b**): *Achillea fragrantissima* (Forssk.); (**c**): *Phoenix dactylifera* (L.).

The *Micromeria fruticosa* (L.) and *Achillea fragrantissima* (Forssk.) Sch.Bip species were collected from the Hebron region in Palestine, while *Phoenix dactylifera* (L.) was collected from the Jericho region in Palestine (Figure 5). The botanical identification of the species was made at the LBEAS laboratory, Biology Department, Faculty of Science, Dhar el Mahraz University, Sidi Mohammed ben Abdallah, Fes, Morocco. The plants are classified in the Herbarium under the voucher numbers MF1/03-11-18/HI; AF2/03-11-18/HI, and PD3/03-11-18/HI. The pollen of the flowers of these plants was then collected manually and dried in the shade. 

### 4.2. Extracts Preparation

One gram of each pollen was macerated in 10 mL of distilled water: ethanol (30:70) for 7 days. After that, they were sonicated (5 min at 20 °C) and centrifuged (5 min at 2000× *g* (rpm)); the supernatants were removed and kept at −20 °C until further use.

### 4.3. Phytochemical Screening of Pollen Extracts of Selected Species from Palestine

Phytochemical screening tests are based on colorimetric reactions and precipitation, and the results are classified according to the appearance into frankly positive reaction: +++; positive reaction: ++; moderately positive reaction: + and negative reaction: −.

#### 4.3.1. Test for Tannins

A volume of 1 mL of the extract was added to 1 mL of 1% FeCl_3_. In the presence of tannins, a greenish or blackish-blue color develops [53].

#### 4.3.2. Test for Catechin Tannins

An extract volume of 5 mL was added to 1 mL of concentrated hydrochloric acid, then the whole was boiled for 15 min. In the presence of catechin tannins, a red precipitate is formed which is soluble in amyl alcohol [53].

#### 4.3.3. Test for Gallic Tannins

An extract volume of 30 mL was added to 15 mL of Stiasny reagent, heated in a water bath for 15 min. Then, the precipitate was filtered and the saturate filtrated with 5 g of powdered sodium acetate. Finally, 1 mL dropwise of FeCl_3_ (1%) solution was added. If precipitates are found, this indicates the presence of gallic tannins [53].

#### 4.3.4. Test for Flavonoids

An extract volume of 1 mL was added to 1 mL of hydrochloric acid, and then a few magnesium shavings and 1 mL of isoamyl alcohol were added. The appearance of an orange pink color indicates the presence of flavones, a purple rose color indicates the presence of flavonones and a red color indicates the presence of flavonols and flavanols [53].

#### 4.3.5. Test for Sterols (Shalkowski Test)

A volume of 1 mL of H_2_SO_4_ was added to the extract. In the contact area of the two liquids there is the formation of a brownish red ring, revealing the presence of sterols [54,55].

#### 4.3.6. Test for Alkaloids

Dragendorff’s reagent (1 mL) was added to 1 mL of each extract. The formation of a cloudy orange color indicates the presence of alkaloids (Dragendorff test).

Mayer’s reagent (1 mL) was added to 1 mL of the extract. The formation of a soft yellow color indicates the presence of alkaloids (Mayer test) [56].

#### 4.3.7. Test for Saponosides

An extract volume of 0.2 mL was added to 5 mL of distilled water, and shaken vigorously for 5 min. The persistence of foams is an indicator of saponosides [56].

#### 4.3.8. Test for Oses and Holosides

Oses and holosides screening was done following the method of Tamert et al. [57]. Two to three drops of concentrated sulfuric acid were added to 1 mL of each extract; after 5 min we added 3 to 4 drops of ethanol saturated with thymol. Appearance of a red color reveals the presence of oses and holosides.

#### 4.3.9. Test for Mucilage

Mucilage was detected by the formation of a fluffy precipitate as described by Tamert et al. [57]. In brief, 5 mL of absolute ethanol added to 1 mL of each extract.

#### 4.3.10. Test for Cardiac Glycosides

An extract volume of 1 ml was added to 2 mL of chloroform. The appearance of a reddish brown color after the addition of H_2_SO_4_ indicates the presence of cardiac glycosides [54,55].

### 4.4. Antioxidant Potential of Pollen Extracts of Selected Species from Palestine

#### 4.4.1. Total Phenolics Content Determination

A volume of 500 μL of Folin-Ciocalteu phenol reagent (10%) was added to 50 µL of each dilution extract. Then, 400 µL of the aqueous solution of Na_2_CO_3_ (0.7 M) was added [58]. The solution was incubated for 2 h. Then, the absorbance was estimated by spectrophotometer at 760 nm. The expression of the total phenolic was recorded as milligrams of gallic acid equivalent per frame of the dry mass (mg GAE/g).

#### 4.4.2. Total Flavone and Flavonol Content

A volume of 500 µL of each extract was mixed with 500 µL of aluminum chloride (AlCl_3_ 20%) [59]. After an incubation of 1 h (at room temperature), the absorbance was measured at 420 nm. The flavone and flavonol content expression was reported as milligrams of quercetin equivalent per gram of the dry mass (mg QE/g).

### 4.5. Antioxidant Activity of Pollen Extracts of Selected Species from Palestine

#### 4.5.1. Scavenging 1,2-Diphenyl-1-Picrylhydrazyl (DPPH) Radical Capacity

The scavenging activities of the prepared pollen extracts were tested against 2,2-diphenyl-1-picrylhydrazyl radical and then estimated following a modified version of the protocol of Brand-Williams et al. [60]. Briefly, 175 μL of DPPH solution was combined with 25 μL of different dilutions of pollen. Mixtures were shackled and then incubated in a dark area for one hour at room temperature. The absorbance of the mixture was observed at 517 nm on a spectrophotometer. The scavenging activity was expressed as a percentage of inhibition using the following equation:

% Inhibition = ((absorbance (control) − absorbance (sample))/absorbance (control)) × 100.

The IC50 (concentration inhibiting 50% of the radicals) was calculated from the graph of inhibition percentage plotted against pollen concentrations using the following equation: IC50 = (50 − b)/a.

a: is the slope;

b: is the y-intercept.

#### 4.5.2. The Reducing Power Assay (FRAP)

The reducing power of the prepared pollen extracts was tested to estimate their potential to reduce ferric ion (Fe^3+^) to ferrous ion (Fe^2+^) following a modified version of Daraghmah and Imtara]’s protocol [6]. In short, 25 μL of different pollen dilutions were combined with a potassium buffer of 200 μL (0.2 M, pH 6.6) and a potassium hexacyanoferrate of 200 μL (1%). The mixture was incubated for 20 min at 50 °C, during which 200 μL (10 percent) of trichloroacetic acid, 600 μL of distilled water, and 120 μL (0.1 percent) of ferric chloride were added. The mixtures were well stirred, and at 700 nm the absorbance was measured. 

The concentration providing 50% radical inhibition (IC50 = mg/mL) was calculated from the graph of absorbance plotted against pollen concentrations using the following equation:

IC50 = (0.5 − b)/a

a: is the slope;

b: is the y-intercept.

### 4.6. Antibacterial Activity of Pollen Extracts of Selected Species from Palestine

#### 4.6.1. Bacterial Strains and Inoculums Standardization

In this study, two Gram-negative strains (*Pseudomonas aeruginosa* and *E. coli* (ATB: 57) B6N) and two Gram-positive strains (*Staphylococcus aureus* and *Streptococcus faecalis*) were used. They were isolated from the Laboratory of Microbiology at the university hospital Hassan II in Fez. The resistance profile of the used bacteria was determined as follows: *E. coli* (ATB: 57) resistance to Cephalothin, Cefotaxime, Ciprofloxacin, Amoxicillin, Trimethoprim-sulphameth-oxazole, and Cefuroxime; *P. aeruginosa* resistant to Trimethoprim-sulphameth-oxazole and Amoxicillin; *S. faecalis* resistant to Trimethoprim-sulphameth-oxazole, Tetracycline, Vancomycin, Oxacillin, Penicillin and Erythromycin, and *S. aureus* resistant to Vancomycin action.

The cultures were held in the refrigerator (4 °C) on Muller-Hinton agar. Colonies from 24-h cultures were used to make the inoculum suspension. The colonies were shacked for 15 s after being suspended in sterile saline (0.9 percent NaCl). The turbidity of a 0.5 McFarland Norm (equivalent to 1–5 108 CFU/mL) was used as density setting.

#### 4.6.2. Agar Well Diffusion (AWD) Assay

Whatman paper discs (6 mm) were inoculated with suspensions (108 CFU/mL) and scattered on the surface of Mueller-Hinton agar plates [7]. Then, with 10 μL of pollen samples, the discs were impregnated. All the plates were incubated for 24 h at 37 °C. The diameters of the inhibition zones were determined after incubation.

#### 4.6.3. Minimal Bactericidal Concentration (MBC) and Minimum Inhibitory Concentration (MIC)

In microplate wells (96 wells), 10 μL of each dilution of pollen extracts varying from 100 mg/mL to 0.20 mg/mL was combined with Mueller Hinton broth (170 μL) and bacterial inoculums (20 μL) and set to a final microbial concentration of 5 × 10^5^ CFU/mL according to NCCLS standards methods [61]. The ethanol content in each well was less than 3.5% in an overall amount of 200 μL. For the negative control, the same percentage of ethanol was used. After 20 h of incubation at 37 degrees Celsius, 40 L of triphenyl tetrazolium chloride was applied to each well. The MIC is the lowest concentration that does not emit a red color after 2 h of incubation. To assess MBC, a portion of each well where the concentrations are > or = (MIC) was sub-cultured on Muller-Hinton agar (MHA) and incubated for 24 h at 37 °C. The MBC is described as the extract concentration at which 99.9% of the inoculated bacteria were destroyed [34].

### 4.7. Statistical Analysis

For the correlation study, the r-value of Pearson correlation was determined between the activities and antioxidant potential (Flavone, flavonol and total phenolic content). The obtained r values were grouped using the dendrogram function of MATLAB 2018a software and presented as a heatmap of three colors: red (r = −1), white (r = 0) and blue (r = 1). Comparisons of pollen extracts were performed by Tukey post-hoc test using SPSS 23 software.

## 5. Conclusions

The present study will be adding its grain of sand to the study of traditional Palestinian medicine. The inclusion of phytochemicals such as alkaloids, flavonoids, sterol, tannins, and cardiac glycosides were reported in the three pollen extracts analyzed. Among them, the pollen extract of *Micromeria fruticosa* showed the highest content of bioactive compounds and has the best antioxidant and antibacterial activities, while the pollen extract of *Phoenix dactylifera* had the lowest content of bioactive compounds and antioxidant activities. The results of this study show that the pollen of these species may be used as a safe substitute for food additives and as an effective treatment for diseases caused by antibiotic-resistant bacterial strains and diseases caused by free radicals. 

## Figures and Tables

**Table 1:** Phytochemical screening of pollen extracts of selected species from Palestine.

Assay			Species	
		*Micromeria fruticosa*	*Achilleafra grantissima*	*Phoenix dactylifera*
Tannins		+++	+++	+++
Catechin tannins		++	+++	−
Gallic tannins		+++	++	+
Flavonoids		+++ (red = flavonols, flavanols)	+++ (orange-pink = flavones)	+++ (purple rose = flavonones)
Sterol		+++	+++	++
Alkaloids	Dragendorff test	+++	++	+
	Mayer test	−	−	−
Saponosides		+	+	−
Cardiac glycosides		+++	+++	+
Oses and holosides		++	+++	−
Mucilage		−	−	−

+: Presence; −: Absence.

**Table 2:** The antioxidant potential of pollen extracts from selected Palestine plants.

	IC50 (mg/mL)	
Species	DPPH (1,2-Diphenyl-1-Picrylhydrazyl)	FRAP
*Micromeria fruticosa*	0.047 ± 0.003	0.039 ± 0.0003
*Achillea fragrantissima*	0.295 ± 0.002 ^a^	0.248 ± 0.007 ^a^
*Phoenix dactylifera*	1.820 ± 0.027 ^a, b^	0.585 ± 0.026 ^a, b^
BHT (butylated hydroxytoluene)	0.061 ± 0.0002 ^b, c^	-
Ascorbic acid	-	0.09 ± 0.0001 ^a, b, c^

Values are expressed as means ± SD. ^a^ *p* < 0.05 as compared to pollen extract of *Micromeria fruticosa* in the same column; ^b^ *p* < 0.05 as compared to pollen extract of *Achillea fragrantissima* in the same column; ^c^ *p* < 0.05 as compared to pollen extract of *Phoenix dactylifera* in the same column.

**Table 3:** Inhibition zones (mm) diameters produced by pollen extracts in an Agar Well Diffusion (AWD) test against Gram positive and negative strains.

Bacterial Strains	*Micromeria fruticosa*	*Achillea fragrantissima*	*Phoenix dactylifera*
*E. coli* (ATB: 57)	13.66 ± 1.5	ND	ND
*P. aeruginosa*	15.66 ± 1.5	15.33 ± 1.15	14 ± 1
*S. aureus*	14.33 ± 0.6	16.33 ± 0.6	ND
*S. faecalis*	16.33 ± 1.5	14.33 ± 1.15	14.66 ± 0.6
Ethanol (70%)	ND	ND	ND

Values are expressed as means ± SD; ND: not determined.

**Table 4:** Minimal inhibitory (MIC) and minimal bactericidal (MBC) concentrations (mg/mL) of pollen extracts of selected species from Palestine.

Species	*Escherichia coli* (ATB: 57)		*Pseudomonas aeruginosa*		*Staphylococcus aureus*		*Streptococcus faecalis*	
	MIC	MBC	MIC	MBC	MIC	MBC	MIC	MBC
*Micromeria fruticosa*	10	>10	5	>10	5	5	0.625	0.625
*Achillea fragrantissima*	10	>10	10	>10	5	10	1.25	1.25
*Phoenix dactylifera*	10	>10	2.5	10	5	10	0.015	0.313
Ethanol (70%)	−	−	−	−	−	−	−	−

−: Not effective.

## Data Availability

Data are available from the authors upon reasonable request.
